# Clinical, Ultrasonographic, Bacteriological, Cytological and Histopathological Findings of Uterine Involution in Ewes with Uterine Infection

**DOI:** 10.3390/pathogens9010054

**Published:** 2020-01-10

**Authors:** Katerina S. Ioannidi, Natalia G. C. Vasileiou, Marianna S. Barbagianni, Denise C. Orfanou, George Mantziaras, Thomas M. Chouzouris, Eleni Dovolou, Dimitris C. Chatzopoulos, Emmanouil Karavanis, Nikolaos Papadopoulos, Angeliki I. Katsafadou, Ilektra A. Fragkou, Nikos G. Kordalis, George S. Amiridis, George C. Fthenakis, Vasia S. Mavrogianni

**Affiliations:** 1 Veterinary Faculty, University of Thessaly, 43100 Karditsa, Greece; kate_ioan@windowslive.com (K.S.I.); vasileiounat@gmail.com (N.G.C.V.); mabarbag@vet.uth.gr (M.S.B.); dorfanou@vet.uth.gr (D.C.O.); tmchouzouris@gmail.com (T.M.C.); lena.dovolou@yahoo.gr (E.D.); vetdchatzop@gmail.com (D.C.C.); agkatsaf@vet.uth.gr (A.I.K.); hfragou@vet.uth.gr (I.A.F.); nikolaoskordalis@gmail.com (N.G.K.); gsamir@vet.uth.gr (G.S.A.); vmavrog@vet.uth.gr (V.S.M.); 2 Veterinary Department, Hellenic Air Force General Staff, 11525 Athens, Greece; gmantziaras@yahoo.com; 3 Histopathology Laboratory, 3rd Veterinary Hospital of Greek Army, 57000 Thessaloniki, Greece; ekaravanis@gmail.com (E.K.); npapadovet@yahoo.gr (N.P.)

**Keywords:** Doppler, endometritis, metritis, pregnancy toxaemia, reproductive performance, sheep

## Abstract

The objectives of the study were (a) to study the characteristics of uterine involution in ewes that had developed subclinical uterine infection in the immediately post-partum period and (b) to evaluate effects of the infection in the subsequent reproductive performance of ewes. Uterine infection was induced in ewes (I, n = 10) by intrauterine inoculation of *Escherichia coli*; uninoculated controls were included (C, n = 12). Animals were examined at regular intervals before and post-inoculation. Clinical and ultrasonographic examinations were performed. Vaginal swab samples and biopsy uterine tissue samples were collected for bacteriological, cytological and histological examination. Finally, ewes were put to rams and reproductive performance was monitored. After challenge, it was ultrasonographically found that caruncular dimensions, myometrial thickness and diameter of uterine lumen were greater in I ewes. In these ewes, particular reduction of dimensions occurred during the second week post-partum, whilst in C ewes during the first week. The uterine artery diameter and the blood flow into the uterus were also greater in I than in C ewes. *E. coli* infection was more frequent and of longer duration in I than in C ewes: in 68.1% and 50.0% of ewes and 19.5 and 14 days, respectively. There was lower proportion of neutrophils and higher of lymphocytes in group I than in C. In inoculated ewes, there was histological evidence of uterine epithelial destruction, increased cellular infiltration, hyperaemia and extracasation, which persisted up to 42 days post-partum. During the subsequent reproductive season, all ewes in group I lambed normally and produced healthy and viable lambs. No significant difference in reproductive performance parameters were seen in I comparison to C ewes. It is concluded that the innate immunity of the uterus sufficed to counteract the bacterial infection, although the process of involution took longer than in healthy animals; moreover, the ultrasonographic examination is a useful means for assessment of the genital tract of ewes post-partum; finally, no adverse effects were noted in the subsequent reproductive performance of ewes.

## 1. Introduction

The puerperium is the period after completion of parturition. The period includes many progressive changes in the genital tract for returning to normal pre-gravid state. According to Noakes [1], in ewes the reduction of the size of the genital tract occurs at logarithmic scale, especially during the first week after lambing. During the 3rd to 10th day post-partum, a rapid reduction in the size of the uterus occurs, which coincides with a decrease in frequency and duration of uterine contractions [1]; other researchers [2,3] have indicated that 50% of uterine reduction was achieved within two weeks after lambing. According to Fernandes et al. [4], the reduction in uterine size continued until the 21st day post-partum, but at a slower rate; then, on the 28th day post-partum, the uterine body diameter was found to be approximately 2 cm. In general, most relevant studies conclude that uterine involution is complete by the 35th day post-partum (reviewed by Ioannidi et al. [5]).

In ewes, post-partum uterine infections have not been studied as extensively as the respective problems in cows [6]. Although they occur less frequently than in cows, they can still be a cause of periparturient death in sheep [7]. Metritis is an opportunistic infection of the uterus, usually caused by contaminant environmental bacteria [8]. Various bacteria may act as causal agents, with *Escherichia coli* and *Trueperella pyogenes* being the most frequent ones; less frequently, staphylococci, streptococci, *Pseudomonas aeruginosa*, *Klebsiella* spp., *Proteus* spp. and anaerobic bacteria are involved [9,10]. There are few studies of the infection in ewes, most of them related to reports about spontaneous cases of the disorder; moreover, its potential impact in the subsequent reproductive performance of ewes has not been reported. Uterine infections can usually develop as the consequence of obstetrical manipulations (commonly unskilled or unhygienic), retention of foetal membranes, delivery of dead lambs and uterine prolapse [8].

The objectives of the present work were (a) to study the characteristics of uterine involution in ewes that had developed uterine infection in the immediately post-partum period and (b) to evaluate effects of the infection in the subsequent reproductive performance of ewes.

## 2. Results

### 2.1. Clinical Findings

In group I, all 10 ewes developed uterine infection after challenge. One ewe (0.100, 95% CI: 0.018–0.404) developed transiently increased rectal temperature (40.9 °C for 1 day). All ewes (1.000, 95% Confidence Interval [CI]: 0.723–1.000) developed genital clinical signs (presence of malodorous, thick, purulent, yellow- to brown- to black-coloured vaginal discharge [n = 7] and vaginal hyperaemia and vulval oedema [n = 7]) within one day post-challenge. Median duration of genital clinical signs was 29.5 (19.5–61) days. Recurrence of clinical signs was recorded in three ewes (0.300, 95% CI: 0.108–0.603).

In group C, systemic signs were not observed in any ewe. In 2 ewes (0.167, 95% CI: 0.047–0.448), there was transient presence of vaginal discharge; median duration of its presence was 11 (9–13) days. No recurrence occurred in these ewes.

Frequency of uterine infection was significantly higher in group I (*p* = 0.0001). Inoculation increased the risk of development of infection; odds ratio was 88.200 (95% CI: 3.762–2067.754) (*p* = 0.005). Median duration of clinical signs was significantly longer in group I (*p* = 0.025).

### 2.2. Ultrasonographic Findings

#### 2.2.1. B-Mode Examination

**Figure 1:**
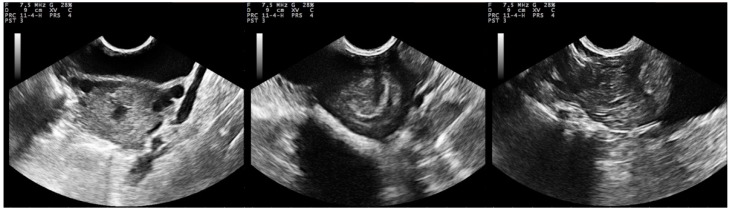
Sequential post-partum B-mode ultrasonographic presentation of the uterus of ewes with experimentally induced uterine infection (group I), **left to right**: immediately after lambing, D2 and D12; uterine body and lumen are imaged (transverse sections obtained by transcutaneous examination at the inguinal area, image taken and processed on a MyLab^®^ 30 ultrasonography system (MyLab^®^ 30; ESAOTE SpA, Genova, Italy) with convex transducer, imaging frequency: 7.5 MHz; scanning depth: 90 mm) (D0: day of inoculation).

In I group ewes, the uterus could be easily imaged during the first week post-challenge, with the endometrium appearing more hyperechoic than in healthy control ewes. The layers of the uterine wall appeared thickened and particularly echogenic until D12. Content of the uterine lumen was imaged with hyperechoic foci, due to the presence of fluid and cellular components in most ewes until D9 (until D17 in two ewes). Progressively, the uterine content was imaged containing inconsistent and blurred structures of increased or mixed echogenicity; the last day that uterine content was imaged was D17. Post-challenge, caruncles were echogenically similar to the endometrium and its folds, but briefly, on the 2nd week post-challenge, they became more echogenic than that (Figure 1). Indeed, during S_3_, GS_C_/GS_E_ ratio was greater in group I than in group C ewes (*p* < 0.001) (Table 1). For all measurements after challenge, GS_C_/GS_E_ ratio was 1.05 ± 0.03 in group I and 0.96 ± 0.03 in group C ewes (*p* = 0.057).

**Figure 2:**
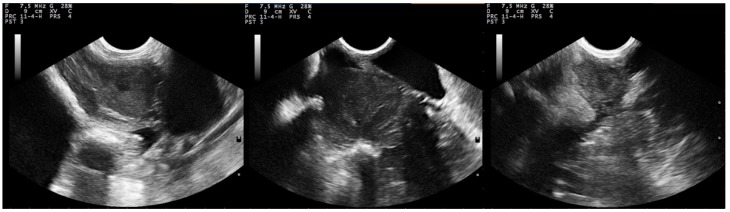
Sequential post-partum B-mode ultrasonographic presentation of the uterus of normally involuting ewes (uninoculated controls) (group C), **left to right**: immediately after lambing, D2 and D12; uterine body and lumen are imaged (transverse sections obtained by transcutaneous examination at the inguinal area, images taken and processed on a MyLab^®^ 30 ultrasonography system with convex transducer, imaging frequency: 7.5 MHz; scanning depth: 90 mm) (D0: day of inoculation).

In group C, layers in the uterine wall were clearly imaged: the perimetrium and the outer layer of myometrium were highly echogenic, the middle layer of myometrium was anechoic to hypoechoic, like a dark rim, inner layer of myometrium and the endometrium appeared as echogenic structures. The uterine lumen was seen irregularly shaped. After the 10th day post-partum, the organ appeared circular or elliptical (longitudinal sections) or polygonal to elliptical, progressively changing to compressed circular to circular (transverse sections). Caruncles were seen on the endometrium, often appearing in mushroom-like shape. In general, they were echogenically similar to the endometrium and its folds (Table 1); differences in their echogenity were evident: the most superficial layer was hyperechoic, with the deeper tissue being less echoic; caruncles were last observed on the 23rd day post-partum. Progressive degeneration of caruncles and removal of endometrium surface led to presence of echogenic content within the anechoic lumen. The uterine body bifurcation and the horns could be imaged up to 33rd day post-partum. Until then, the myometrium and the endometrium were imaged as clearly separate layers. Subsequently, the organ was imaged as a round entity near the bladder, as its size decreased. The echopattern of the uterine wall was finely textured, homogeneous and hypoechoic to moderately echogenic (Figure 2).

After challenge, caruncular dimensions were greater in inoculated ewes (group I) than in controls (group C) (Table 2). Myometrial thickness was also greater in I than in C ewes: 0.318 ± 0.007 cm versus 0.297 ± 0.008 cm (for all measurements taken, *p* = 0.016), as was diameter of uterine lumen: 0.368 ± 0.027 cm versus 0.339 ± 0.013 cm (for all measurements taken, *p* = 0.11).

In all ewes, there was a reduction of dimensions of uterine structures progressively, as the post-partum period advanced. In group I ewes, particular reduction of dimensions occurred during S_3_, when mean daily reduction of the various structures was calculated up to 15.5%. In contrast, in group C ewes, greatest reduction of dimensions occurred during S_2_ (i.e., during the first week after lambing), when daily reduction of dimensions of the uterine structures varied from 4.2% to 9.2% (Table 3). Overall, throughout the monitoring period, there were no significant differences in daily reduction of the various structures between the two groups; for group I, it was 41.2% for caruncular diameter, 50.5% for myometrial thickness, 63.2% for endometrial thickness and 63.3% for uterine lumen diameter, whilst for group C, it was 45.0%, 52.4%, 54.8% and 61.9%, respectively (*p* = 0.39, 0.28, 0.11, 0.40, respectively).

#### 2.2.2. Doppler Mode Examination

**Figure 3:**
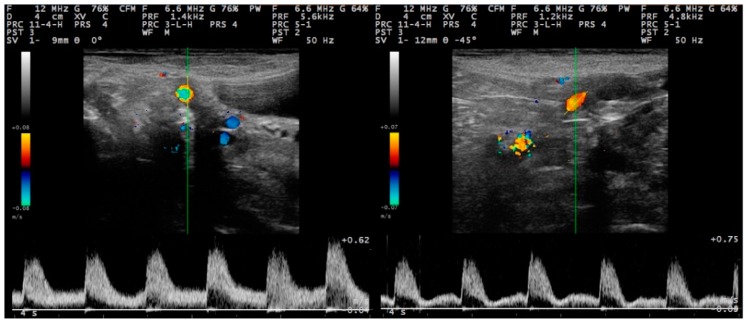
Sequential post-partum spectral waveforms of the uterine artery (Doppler ultrasonography) of ewes with experimentally induced uterine infection (group I), **left to right**: D2 and D12 (images taken and processed on a MyLab^®^ 30 ultrasonography system with linear transducer, Doppler imaging frequency: 6.6 MHz; scanning depth: 40 mm) (D0: day of inoculation).

**Figure 4:**
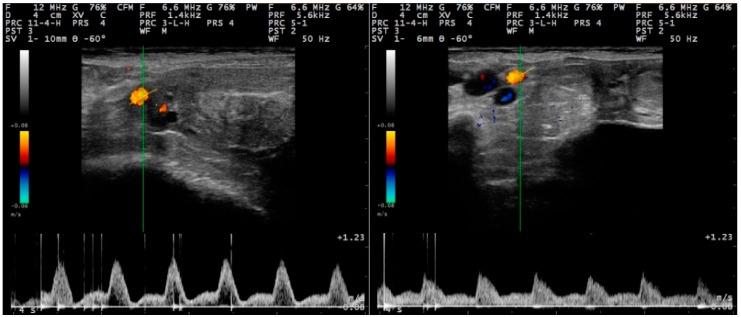
Sequential post-partum spectral waveforms of the uterine artery (Doppler ultrasonography) of normally involuting ewes (uninoculated controls) (group C), **left to right**: D2 and D12 (images taken and processed on a MyLab^®^ 30 ultrasonography system with linear transducer, Doppler imaging frequency: 6.6 MHz; scanning depth: 40 mm) (D0: day of inoculation).

The spectral display in Doppler mode was observed as a broad band structure (Figure 3 and Figure 4). The diameter of the uterine artery was similar in the two groups on S_1_, but thereafter remained significantly larger in group I ewes (*p* < 0.02). The resistance and pulsatility indexes remained smaller in the group I ewes throughout the study, whilst the blood pressure was higher in these ewes. Finally, blood flow volume into the uterus was significantly higher in group I ewes after challenge (*p* < 0.02) (Table 4). Moreover, for both groups, the progressive changes in the haemodynamic parameters (except for blood velocity) were significant (Table 4).

**Figure 5:**
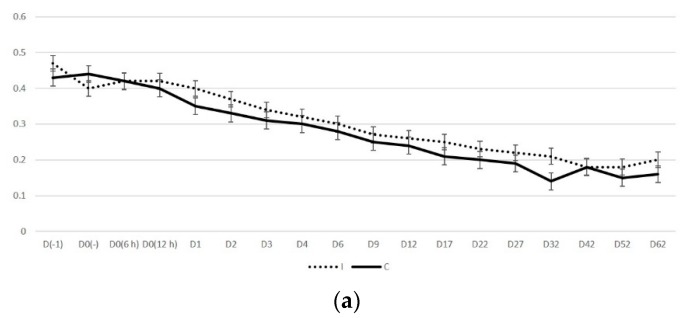

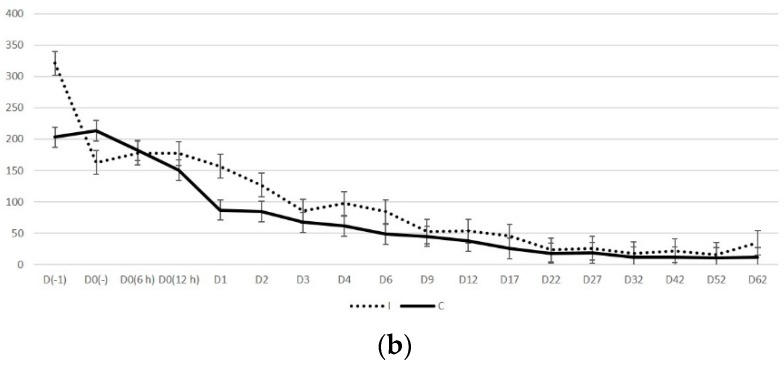
Time-series graphs (D0: day of inoculation) of Doppler ultrasonography results of the uterine artery of ewes with experimentally induced uterine infection (group I) or of normally involuting ewes (uninoculated controls) (group C) (**a**) diameter of the vessel (cm), (**b**) blood flow volume (mL min^−1^).

For all measurements taken after challenge, the uterine artery diameter and the blood flow volume into the uterus were significantly greater in I than in C ewes: 0.31 ± 0.01 cm versus 0.27 ± 0.01 cm (*p* = 0.008) and 92.7 ± 7.2 mL min^−1^ versus 62.0 ± 5.3 mL min^−1^ (*p* = 0.032), respectively (Figure 5). For the other haemodynamic parameters evaluated, no significant differences were evident (*p* > 0.20).

#### 2.2.3. Contrast-Enhanced Examination

**Figure 6:**
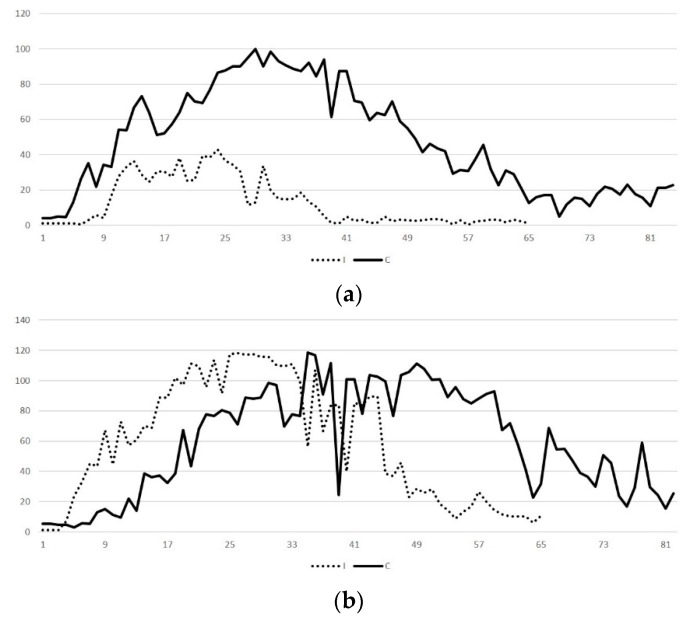
Patterns of image enhancement by contrast-enhanced ultrasonographic examination in the uterus of ewes with experimentally induced uterine infection (group I) or of normally involuting ewes (uninoculated controls) (group C) on D1; (**a**) caruncular tissue, (**b**) intercaruncular area (D0: day of inoculation).

No adverse effects were observed clinically in any animal after administration of the contrast agent. The dose administered allowed clear imaging of the structures in all cases. In healthy ewes, contrast-enhanced ultrasonographic (CEUS) examination revealed a steady biphasic pattern of contrast agent kinetics, characterised by initial uptake (wash-in phase) within 70 s post-injection, at which time intensity peaked with strong enhancement (80–100 AEU for caruncles and 100–120 AEU for intercaruncular areas), followed by a gradual wash-out phase (Figure 6). In contrast, in the uterus of group I ewes, the pattern showed the enhancement being weak in the caruncles (<50 AEU; *p* < 0.001), but similar to that in group C for the intercaruncular area (100–120 AEU; *p* = 0.47) (Table 5).

**Figure 7:**
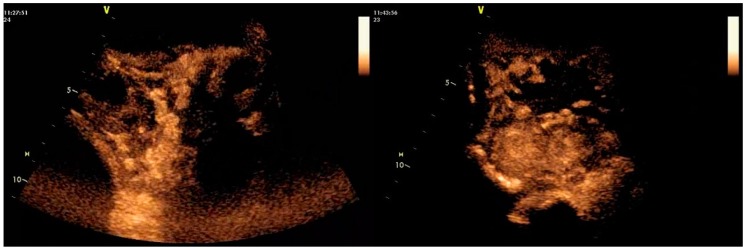
Contrast-enhanced ultrasonographic presentation of uterine caruncles; image taken on D1; left: weak and partial imaging of uterine caruncles of a ewe with uterine infection, with reduced enhancement in 48 s, right: full imaging of uterine caruncles of a healthy ewe (uninoculated control), with peak enhancement in 58 s (images taken and processed on a Vivid-I ultrasonography system with convex transducer, imaging frequency: 2.0/4.0 MHz; mechanical index: 0.09; power: 22dB; scanning depth: 70 mm; contrast agent: 40 μL sulphur hexafluoride in microbubbles) (D0: day of inoculation).

Enhancement and clearance were evident initially in the caruncular tissue. Enhancement in the intercaruncular area revealed an irregular pattern; it started with a delay, but lasted longer than in caruncles. Enhancement allowed clear visualisation of the entire caruncles in the control ewes; in inoculated ewes, caruncles could be visualised only partially (Figure 7).

### 2.3. Bacteriological Findings

#### 2.3.1. Vaginal Swab Samples

In group I, *E. coli* was isolated from all ewes (1.000, 95% CI: 0.723–1.000) after challenge. Median duration of infection was 19.5 (1.5–62) days. Recurrence of infection was noted in 6 ewes (0.600, 95% CI: 0.313–0.832). *E. coli* was isolated in 109 samplings (0.681). In 29 samplings (0.181) other bacteria (staphylococci, streptococci, *Trueperella* spp., Enterobacteriaceae) were isolated, in 12 of these (0.414) in mixed culture with *E. coli*. In total, 142 bacterial isolates were recovered (0.888 isolates per sampling).

In C group, bacteria (*E. coli* or other organisms) were isolated from all ewes (1.000; 95% 0.758–1.000) at least once. Median duration of infection was 14 (1.25–62) days (*p* < 0.001 when compared with group I). Recurrence of infection was noted in 8 ewes (0.800, 95% CI: 0.391–0.862) (*p* = 0.55 when compared with group I). *E. coli* was isolated in 96 samplings (0.500), i.e., less frequently than from I ewes (*p* < 0.001). Other bacteria were isolated in 69 samplings (0.359), i.e., more frequently than from I ewes (*p* < 0.001), in 25 of these (0.362) in mixed culture with *E. coli*. In total, 173 bacterial isolates were recovered (0.901 isolates per sampling) (*p* = 0.34 when compared with group I).

In group I, frequency of *E. coli* isolation was significantly higher in S_2_ and S_3_ than in S_4_ (*p* < 0.001). In contrast, no such difference was seen between stages in group C (*p* > 0.13). Details are in Table 6.

#### 2.3.2. Uterine Samples

In group I, *E. coli* was recovered twice, on the first sampling of the respective animals (0.182), one on D7 and one on D14. In group C, no bacteria were recovered from any uterine sample (0.000) (*p* = 0.43).

### 2.4. Cytological Findings

In vaginal swab samples from all ewes, neutrophils were the predominant leucocyte type observed therein (Table 7). There was a significantly lower proportion of neutrophils in group I (80.5% ± 2.7%) than in group C (91.0% ± 1.8%) ewes; proportion of lymphocytes in group I (7.3% ± 1.3%) increased immediately after challenge and was higher than in group C (3.8% ± 1.0%) (for all measurements taken after challenge, *p* = 0.003 and 0.034, respectively) (Table 7). Further, the progressive changes throughout the study in the proportions of leucocytes (neutrophil reduction, lymphocyte increase) were also significant in both groups.

### 2.5. Haematological Findings

Details of haematological findings are in Table 8. The only parameter in which there was a significant difference between group I and C ewes for all measurements after challenge, was eosinophil counts: 204 ± 10 cells mL^−1^ versus 429 ± 34 cells mL^−1^, respectively (*p* = 0.003). For the other parameters measured, no significant differences were evident (*p* > 0.06).

### 2.6. Histological Findings

**Figure 8:**
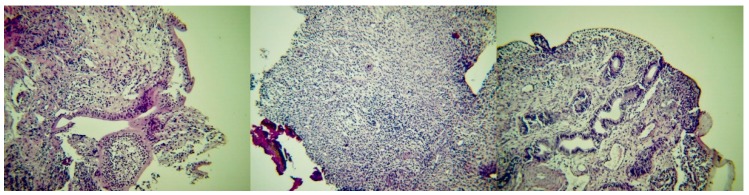
Post-partum histological pictures of the uterus of ewes with experimentally induced uterine infection (group I), **left to right**: D7 (section of the endometrium, with cylindrical-like cells in the epithelium; occasionally, epithelium completely detached from the endometrium; presence of intense subepithelial infiltration primarily by lymphocytes with a few neutrophils as well), D27 (section of the endometrium, with intense infiltration by lymphocytes) and D42 (section of the endometrium, with cyboidal single-layered epithelium in almost the entire length of the endometrium; subepithelial infiltration primarily by lymphocytes; presence of vessels and uterine glands with cylindrical single-layered epithelium) (H & E, 200×).

In group I ewes (Figure 8), in samples collected on D7, the epithelium of the endometrium had a loose structure, with high cylindrical-like cells, which contained vacuoles. There was intense vascularisation with lymphocytic infiltration, which occurred mainly subepithelially and occasionally around the uterine glands. These could be easily observed. The myometrium appeared to have lost its fine structure; hyperaemia and extravasation were evident therein and it also had a loose appearance. On D14, the epithelium of the endometrium appeared destroyed with intense neutrophilic and lymphocytic infiltration; there was intense vascularisation and extravasation. There was intense presence of lymphocytes in the myometrium. On D24 and D27, the epithelium of the endometrium included cylindrical cells, with several small glands with small diameter being clustered in groups; the epithelium of the glands was cuboidal. There was intense presence of lymphocytes in the endometrium, whilst no inflammatory cells were observed in the myometrium, which included intense vascularisation and extravasation. On D34, in the sample from one ewe the epitheliium of the endometrium was characterised by single-layered cells, whilst in the sample from the second animal no epithelium could be observed. The uterine glands were collapsed and had fully regressed. The myometrium was thickened; no cellular infiltration could be observed therein in the sample from one ewes, whilst in the sample from the second animal there was evidence of intense cellular infiltration. On D42 and D44, there were single-layered cells on the epithelium of the endometrium, but this was not absolutely intact, as there were areas in the samples that no epithelium could be seen. Cellular infiltration in the myometrium was still evident. On D62, the border between the endometrium and the myometrium could not be distinguished; there were leucocytes and mild vascularisation therein.

In group C ewes, in samples collected on D7 and D14, the epithelium of the endometrium was intact, with cuboidal- to cylindrical-like cells; these contained vacuoles. There was clear hyperaemia with small-degree neutrophilic infiltration. The uterine glands were observed easily and had also cuboidal- to cylindrical-like epithelial cells. The myometrium included trophoblast-like cells and vacuoles. On D24 and D27, the epithelium was still intact, but height of epithelial cells was decreased. The uterine glands had a smaller size compared to previous samples. Progressively, the thickness of the myometrium reduced. On D34, the epithelium of the endometrium was characterised by single-layered cells. The uterine glands were collapsed and had fully regressed. Leucocytic infiltration was characterised by presence of lymphocytes. On D42 and D44, there were single-layered cells on the epithelium of the endometrium, still with lymphocytic infiltration. Finally, on D62, the border between the endometrium and the myometrium could not be distinguished and the entire uterine wall was observed as one entity.

Median score for total number of leucocytes in samples from I ewes was higher than in C ewes: 4 versus 3 (*p* = 0.014); differences between I and C ewes in median scores for neutrophils and lymphocytes, when these were considered separately, were not significant (*p* = 0.50 and 0.38, respectively). Lymphocytes always predominated in tissue samples, independently of sampling point or group.

### 2.7. Subsequent Reproductive Performance

During the subsequent reproductive (breeding) season, all ewes in group I and group C were mated and diagnosed to be pregnant by using ultrasonographic examination. Finally, all ewes lambed normally and produced healthy and viable lambs. There were no significant differences between groups I and C in any reproductive performance parameter (*p* > 0.17) (Table 9).

## 3. Discussion

During the puerperium, the genital system is returning to its non-pregnant state. Nevertheless, it does not completely return to the original pre-gravid state, as some of the changes taking place during gestation are not completely reversible (e.g., size of the uterus). In this study, we used an established model for inducing uterine infection and its potential effects in uterine regression. Development of the disease was confirmed by means of clinical, bacteriological and cytological findings.

The present results indicated that *E. coli* constituted the major proportion of the bacterial populations of the vagina in inoculated ewes, which may reflect its presence in uterine exudate leaking into the vagina. In these animals, other organisms were also recovered, but they were significantly less frequent than in control ewes. In cases of infections with established pathogens, bacterial flora in affected sites decreases. Antagonism of invading pathogens versus commensal organisms present in the affected body site has been reported in cases of intestinal infections; for example, *Vibrio cholerae* ‘attacks’ members of the gut microbiota, thus facilitating its colonisation of the intestine [11], and *Salmonella typhimurium*, in a T6SS-mediated manner, kills commensal organisms, in order to establish itself in the intestine of affected hosts [12]. As the T6SS nanomachine has also been identified in *E. coli* [13], one cannot rule out that this pathogen in a similar manner possibly limited the populations of genital commensal bacteria in the inoculated ewes, in order to secure its dominance in the infected genital tracts.

Ewes often lamb during the anoestrus period, which does not involve hormones in the genital tract post-partum. In contrast, during the breeding period, when ovulations take place with a subsequent increase of progesterone concentrations, a local immunosuppressive effect on the endometrium can occur, which may increase the risk of bacterial complications during uterine involution [14]. This can be of importance in systems, in which ewes are subjected to reproductive control soon after lambing, to be mated for accelerating production and achieving two lambings within a year.

Post-challenge, lymphocytes predominated in the inoculated ewes, as seen in vaginal and uterine samples. Bacterial phagocytosis by migrating leucocytes is the principal mechanism involved in elimination of intrauterine bacteria [1]. Nevertheless, Cai et al. [15] have reported that phagocytic ability and intracellular bacterial killing capacity of neutrophils in cows with post-partum infections were impaired. Therefore, lymphocytes become of particular value to clear uterine infections. In cows, it has been found that subepithelially located lymphocytes, which were also found in the present study, included CD4+ cells and B lymphocytes [16], playing a paramount role in the clearance of intrauterine bacteria. Brodzki et al. [17] reported that in cows with metritis CD4+ cells were reduced and CD8+ prevailed. Endometrial epithelial cells may also participate in the fight against pathogens by expressing receptors, which would recognise components of bacterial cells (e.g., lipopolysaccharides in the case of *E. coli*) [18]. Hence, the damage in the uterine epithelium, as seen in samples from inoculated ewes, might have contributed in the delayed clearance of the pathogen recorded in the study. In general, uterine defence mechanisms can be impaired during the luteal phase of the ovarian cycle. Hence, in ewes that lamb during the anoestrous period, the cellular mechanisms are effective against intrauterine bacteria, whilst in ewes that would be subjected to reproductive control for accelerated lambing, one may express concerns regarding a possible impairment of these defences.

The importance of lymphocytes in controlling the infection is also underlined by the increased number of these cells in blood. In a previous study, this increase has been found to take place specifically in *vena cava* blood samples [19]. This may reflect a movement of lymphocytes from secondary lymphatic tissue into the blood circulation, whence it can move into the uterus, as identified in relevant samples. The importance of lymphocytes is reflected in the progressive and significant increase of their proportion in genital tract samples, which is compatible with the progressive increase of lymphocyte counts in blood. In infected ewes, the smaller eosinophil counts in blood, present only post-challenge, can be allied to increased presence of these cells in the uterine tissue, which in women has been suggested as a preliminary diagnostic means for long-standing endometritis [20]. In the endometrium, eosinophils possess high affinity receptors for IgE [21] and binding of antibodies to these receptors results in the degranulation of these cells, leading to the release of inflammatory mediators, superoxides, lytic enzymes and kallikreins [21].

Ultrasonographic examination has advantages over other techniques that may be employed for monitoring the genital system, e.g., laparascopy, the applicability of which under clinical conditions is difficult and potentially risky [22]. In contrast, ultrasonographic examination is an easily applied and accurate technique. In cows, the technique is used successfully for the diagnosis of subclinical post-partum uterine infections [23,24]. Kasimanickam et al. [25] concluded that ultrasonographic examination of the genital tract post-partum can be used to evaluate the uterus. Further, ultrasonographic examination of ewes post-partum may be used to investigate potential genital disorders, which do not manifest with striking clinical signs; the method can also be used to assess response to treatment in cases of uterine infections. The transcutaneous technique allowed visualisation of structures (B-mode) and recording of haemodynamic parameters (Doppler mode) throughout the post-partum period; the technique is more animal-friendly and easier to apply than the transrectal technique that has also been used in the same conditions [5].

The results of B-mode ultrasonographic evaluation revealed a delay in the process of involution of the uterus in inoculated ewes. In these animals, the principal regression took place during S_3_, i.e., in contrast to control ewes, in which regression occurred mostly during S_2_. In previous studies, in which ultrasonographic examination was employed to study the genital tract of ewes, uterine involution was found to have been completed within 35 days post-lambing (e.g., [2,22,26,27,28]).

The findings can be associated with the results of histological evaluation of uterine tissue samples. Based on these findings, regression was considered to be complete between D34 and D44 in control ewes (according to day of biopsy-sampling). Gray et al. [29] have defined as a major criterion for regression the eversion of caruncles and the ‘re-epitheliazation’ (*sic*) of the uterus. Although that study did not extend beyond the 28th day post-partum, those authors reported that, on that day, the process was nearing its completion, a conclusion that is in line with the present findings. In contrast, in the inoculated ewes, regression was not fully completed at above days, which indicates a delay in the involution of the organ.

The results of Doppler evaluation further corroborate the delay in uterine involution. In inoculated ewes, the greater diameter of the uterine artery and the increased blood volume were obviously the result of the inflammation [30]. The increased blood flow recorded during the Doppler evaluation is associated with the frequent findings of hyperaemia and extravasation in uterine tissue samples from inoculated ewes.

Nevertheless, at the end of the monitoring period, the uterus in the inoculated ewes had also regressed, as confirmed by the ultrasonographic and histological findings. This is corroborated by the lack of differences between the two groups in the reduction of dimensions for any uterine structure for the entire monitoring period.

Finally, the present results did not show that uterine infection had any adverse consequences in the subsequent reproductive performance of ewes. In fact, all those ewes were mated and lambed as planned. In anoestrous ewes, there is a prolonged period of sexual rest; although there is a minimal follicular development, the reproductive system of ewes is quiescent. One may suggest that the genital tract had recovered during that period, before new exposure to a period of progesterone influence [31]. Potentially, if the ‘resting’ period of the genital tract would be shorter (e.g., as in accelerated production systems in meat-producing flocks), one may postulate that some adverse effects would possibly be noted in the subsequent reproductive performance of the animals.

Under field conditions, in cases of metritis, veterinarians would often prescribe to affected animals a broad spectrum antibiotic, often coupled with a non-steroid anti-inflammatory agent. These actions are in accord with good clinical veterinary practice. In the present study, no therapeutic intervention was performed, as there was interest in following the course of the disease as it developed. The results have indicated that ewes recovered spontaneously, in the absence of a treatment and with no subsequent adverse reproductive effects. This can be taken into account, in order to reduce the use of antimicrobial agents, which would contribute in limiting development of resistance to antimicrobial agents. Nevertheless, each case should be considered individually and appropriate interventions should be made if necessary, having always in mind the welfare of the animals under consideration.

Due to the small number of animals involved and measurements performed, the CEUS work should be considered as preliminary and indicative only. The increased cost of the technique (approx. 100 Euros per animal, per examination) was a limiting factor for furthering the work at this stage. Nevertheless, the work has indicated that the dose of the contrast-agent administered, allowed clear visualisation of the uterine structures. Further, decreased enhancement in the inoculated uteri indicated reduced perfusion of contrast agent into the organ, which occurred despite the increased blood flow therein as the consequence of early stage of inflammation. This may possibly indicate that some tissue damage to the organ has occurred already within 24 h of inoculation.

In conclusion, the innate immunity of the uterus sufficed to counteract the bacterial infection, although the process of involution took longer than in healthy animals. The ultrasonographic examination (B-mode, Doppler mode, CEUS) is a useful means for assessment of the genital tract of ewes post-partum. Finally, no adverse effects were noted in the subsequent reproductive performance of ewes.

## 4. Materials and Methods

### 4.1. Experimental Design

In total, 24 Lacaune-cross ewes (age 3–5 years old) were included in the study. Before enrolment, a standardised detailed clinical examination was performed (authors KSI, NGCV) to assess the general health of the ewes. After enrolment in the study, ewes were identified using neck straps and plastic tags with unique serial numbers. Reproductive control (latitude of location: N 39.37°, month of application: September) was performed by intravaginal insertion of progestogen sponges. Ewes were mated by rams of known fertility and repeatedly examined ultrasonographically to confirm pregnancy and its normal progress [32]. Standard health management procedures were performed in the animals during gestation [33].

Two weeks before the expected lambing date, animals were allocated in two equal groups (I and C) by using complete randomisation, through random number generator. After allocation into groups, all animals in the same group were penned together and separately from the other group.

All ewes lambed normally (L0 = day of lambing) and produced twin lambs. A detailed clinical examination was performed immediately post-partum. Uterine samples were collected as described below (Section 4.2); two animals in group I that yielded *E. coli* in those samples were excluded from the study. The remaining 10 ewes in group I were challenged on the 1st day post-partum (L1, D0 = day of inoculation) with approximately 3.5 × 10^5^ colony-forming-units of an *E. coli* isolate originally from a field case of metritis, inoculated into the uterus of each of these ewes. In ewes in group C, sterile phosphate buffer saline pH 7.3 (PBS) was injected into the uterus.

The challenge procedure was as follows [34,35]. Two colonies of the isolate obtained from a fresh culture, were inoculated into 10 mL of Soy-broth (BioMerieux, Marcy-l’ Etoile, France) and incubated aerobically at 37 °C. After 6 h incubation, serial dilutions of the broth into PBS were carried out and 1.0 mL of the desired dilution was centrifuged for 10 min at 1750× *g*. The supernatant was discarded and the bacteria resuspended in approximately 2.0 mL sterile PBS and transferred into 5 mL syringes. The number of viable bacteria in the suspension was counted by the method of Miles and Misra [36]. Each syringe was attached to the end of a sterile catheter, which was inserted into the uterus of the ewe, through the cervix. The inoculum was deposited at the body of the uterus.

Conditions prescribed by legislation of the European Union in relation to animal experimentation procedures (Council Directive 86/809/EEC) were met during this work. Licence no. 2542/97549 was issued by the local veterinary authority to allow the experimentation.

### 4.2. Post-Partum Examinations and Sample Collection

Detailed clinical examinations of the genital tract of all ewes were performed at regular intervals throughout the study. Before challenge, two samples were collected, one immediately after lambing (L0) and the second on the following day (L1). After inoculation, samples were collected initially 6 h and 12 h post-challenge (D0 + 6 h and D0 + 12 h, respectively) and then on D1, D2, D3, D4, D6, D9, D12, D17, D22, D27, D32, D42, D52, D62.

On these sampling points, samples of vaginal discharge were also collected. Initially, thorough cleansing of the external genitalia was performed with povidone iodine scrub solution (Betadine^®^; Mundipharma Medical Company, Basel, Switzerland). Then, a sterile swab was introduced into the vagina, through a plastic, single-use speculum and a long, lubricated, sterile protective sheath, in order to sample any discharge present at the outer entrance of the cervix and the anterior part of the vagina; the wall of the genital tract was gently swabbed and the swab was withdrawn. Collection of samples for cytological examination was performed always by the same person (author KSI). Thereafter, a cell collector with a gentle-touch tip designed to minimise trauma to the genital tract (Cytobrush Plus; Cooper Surgical, Trumbull, USA) was inserted into the genital tract for cell collection. In addition, blood samples were collected on these occasions for standard haematological examination.

Ultrasonographic examination of the genital tract was performed on the above occasions using an ultrasound scanner (MyLab^®^ 30; ESAOTE SpA, Genova, Italy). For B-mode ultrasonography, linear (7.5–12.0 MHz) and convex (2.5–7.5 MHz) transducers were used; the methodology and technicalities described in detail by Ioannidi et al. [17] were followed; longitudinal and transverse sections were taken by means of the transcutaneous technique. For Doppler ultrasonography, a linear (6.6 MHz) transducer was used; the methodology and technicalities described in detail by Ioannidi et al. [5] and Petridis et al. [37] were followed; the transcutaneous technique was used.

Contrast-enhanced ultrasonographic (CEUS) examination was performed on D1 in two group I and two group C ewes, using an ultrasound scanner (Vivid-I; General Electric, Tirat Carmel, Israel), with a convex transducer (4C RS) of varying frequencies (1.8–6.0 MHz); initially, B-mode sections were taken using a frequency of 5.0 MHz and a scanning depth of 120 mm, eventually switching the imaging settings to a preset coded phase inversion mode; frequency, mechanical index and power were automatically set to lower values (i.e., 2.0/4.0 MHz, 0.09 and 22 dB, respectively); one focal zone was used at a scanning depth of 70 mm. A volume of 5.0 mL of the contrast agent (40 μL of sulphur hexafluoride in microbubbles, equivalent to 112.5 mg; excipients: macrogol 4000, distearoylphosphatidylcholine, dipalmitoylphosphatidylglycerol sodium, palmitic acid; solvent: sodium chloride 9 mg mL^−1^) was injected into the jugular vein, followed by intravenous injection of 10 mL of normal saline. This is a second generation contrast agent consisting of microbubbles, containing sulphur hexafluoride, which is an inert and hydrophobic gas, stabilised by a thin and flexible monolayer shell of phospholipids (SonoVue^®^, Bracco, Milano, Italy).

Biopsies were performed laparoscopically (250.2 H; Schoelly Fiberoptic, Denzlingen, Germany) for uterine tissue sample collection from the uterus of the experimental ewes. These were performed on D7, D14, D24, D42 (left horn) and D27, D34, D44, D62 (right horn) (2 animals from group I and 1 animal from group C on each sampling point; biopsy was performed in each animal twice, once in the left and once in the right horn). The animal was placed in dorsal recumbency. Analgesic procedures, with administration of lidocaine 2% (Xylocaine 2%, Astra Zeneca, Cambridge, United Kingdom) were applied [38] and a strict aseptic technique was used. An uterine tissue sample was collected from the respective uterine horn on each occasion as above, by using laparoscopy biopsy forceps (Richard Wolf, Knittlingen, Germany).

### 4.3. Laboratory Examinations

Vaginal swab samples were processed soon (<10 min) after collection. Swabs were cultured on 5% sheep blood agar and McConkey plates. Media were incubated aerobically and anaerobically at 37 °C for up to 48 h; if no bacterial growth was evident, they were reincubated for another 24 h. Bacterial identifications were performed by using standards methods [39,40]. Cells from cell collectors were transferred on glass slides and stained with the Giemsa technique.

Samples for haematological examination were mixed by gentle repeated inversions for several seconds to avoid coagulation and were processed within 30 min. after collection. Initially, blood smears were prepared and kept dry at room temperature. A complete blood count was performed by an automated haematological analyser (ADVIA 2120i; Siemens Healthineers, Erlangen, Germany). The following parameters were determined: erythrocyte count, haematocrit, haemoglobin concentration, mean corpuscular volume, mean corpuscular haemoglobin concentration, total leucocyte count and thrombocyte count. Blood smears were evaluated for detection of morphological abnormalities and leucocyte type differentiation. From proportion of the various leucocyte types, the respective counts were then calculated.

Uterine tissue samples were initially cultured on 5% sheep blood agar and McConkey plates, which were incubated as above. Then, the tissue samples were fixed in 10% neutral-buffered formalin; finally, haematoxylin and eosin (HE) standard staining procedures were used for histological evaluation.

### 4.4. Evaluation of Subsequent Reproductive Performance

Two months after end of the monitoring period and after the reproductive season (i.e., in June of the following year) had started, ewes were put with rams of known fertility (n = 2) for mating. Rams were left with ewes for 60 days. Ultrasonographic examinations were performed for pregnancy diagnosis. Throughout that period, during the pre-conception period and gestation, appropriate health management of ewes had been performed as recommended [33]; all ewes were maintained under the same conditions. Finally, lambings and number of lambs born from each ewe were recorded.

### 4.5. Data Management and Analysis

#### 4.5.1. Post-Partum Stages

The post-partum period was divided into four stages: S_1_ included samples collected before inoculation, on L0 and L1 (2 sampling points), S_2_ included samples collected after challenge up to and including D4 (L5) (6 sampling points), S_3_ included samples collected from D6 to D12 (3 points) and S_4_ included samples collected from D17 to D62 (7 points).

#### 4.5.2. B-Mode Ultrasonographic Measurements

Initially, stored ultrasonographic images were viewed for description. Then, images of (a) caruncular tissue and (b) endometrium obtained from each ewe on each occasion, were processed by means of ImageJ software (National Institutes of Health, Rockville Pike, USA), which can edit, process and analyse grey-scale images, by calculating area and pixel value statistics to produce intensity values [41]; in an image processing context, grey-scale analysis refers to the image’s overall pixel grey intensity values [42], with results expressed on a 0 (black) to 255 (white) scale.

For analysis of results of grey-scale measurements, data were normalised by calculating the ratio GS_C_/GS_E_, where GS_C_: Grey-scale of caruncular tissue and GS_E_: Grey-scale of endometrium.

Measurements of dimensions of uterine structures were performed in images taken by the linear transducer and were calculated by the equipment’s software. For calculation of the diameter of caruncles and the uterine lumen, a measurement of opposing points of the section of the structure was taken, followed by another one at an angle of 90 ^o^ to the first and the mean value of the two was calculated. Thickness of the myometrium and the endometrium was also measured.

Reduction of dimensions of uterine structures (R_s_) within a stage (S_n_) compared to those in the preceding stage (S_n–1_) was calculated as follows for each animal into the study: R_s_ = 1 − (average of measurements taken within S_n_/average of measurements taken within S_n–1_). Daily reduction of dimensions (dR_s_) within a stage (S_n_) was calculated as dR_s_ = [R_s_/(‘mid time-point’ of S_n_ − ‘mid time-point’ of S_n–1_)] (expressed in days), where ‘mid time point’ of S_i_ = [(last day of samplings made during S_i_ − first day of samplings made during S_i_) / 2] + first day of samplings made during S_i_ (last day of samplings: L1, D4, D12, D62 − first day of samplings: L0, D0 + 6 h, D6, D17, for S_1_, S_2_, S_3_, S_4_, respectively). Overall (throughout the study period) reduction of dimensions (R_o_) was calculated as follows for each animal into the study: R_o_ = 1 − (average of measurements taken within S_4_/average of measurements taken within S_1_).

#### 4.5.3. Doppler Mode Ultrasonographic Measurements

Stored images of cross-sections of uterine artery were processed by means of MyLab^®^ software (ESAOTE SpA), which, after pointing out the internal boundaries of the vessel, calculated the internal diameter of the vessel. Results were expressed as cm.

Spectral waveforms of the uterine artery were processed by means of MyLab^®^ software (ESAOTE SpA). On each occasion, waveforms from three consecutive cardiac cycles of the animal under examination were considered for calculations. The software, based on the outline of the waveform, calculated directly the below haemodynamic parameters in that vessel [39,43,44,45].

- Resistance index: [(PSV − EDV)/PSV] (PSV: peak systolic velocity, EDV: end diastolic velocity) indicating the effect of the vessel resisting blood flow.
- Pulsatility index: [(PSV − EDV)/TAMV] (TAMV: time-averaged maximum velocity) measuring the systolic-diastolic differential of the velocity pulse.
- Systolic:diastolic velocity ratio: [ASF/ADF] (ASF: average diastolic flow, ADF: average systolic flow) delineating systolic and diastolic phases of a flow waveform.
- General pressure: [P_syst_ − P_diast_] ([P_syst_: systolic pressure, P_diast_: diastolic pressure) measuring the change in pressure from the diastolic level to the systolic level (mm Hg).
- Mean pressure: [(⅓ × P_syst_) + (⅔ × P_diast_)] measuring the average blood pressure over time by proprietary pulse dynamics pattern-recognition algorithms (mm Hg).
- Mean velocity: indicating blood speed across the vascular lumen at a given instance (m s^−1^).
- Systolic acceleration: indicating blood acceleration across the vascular lumen (m s^−2^).
- Blood flow volume: indicating the volume of blood entering the uterus per unit of time (mL min^−1^).

#### 4.5.4. Contrast-Enhanced Ultrasonographic Measurements

Video images were analysed in sequence of frames (JPG format; first frame at time 0 and then one frame every 2 s) using the Free Studio (v. 6.6.35.323) multimedia software developed by DVDVideoSoft (Digital Wave Ltd, London, United Kingdom). The frames were opened as a stack with ImageJ software. Two regions of interest were used in the evaluation: caruncular tissue and intercaruncular area for calculation of intensity of signals. Image enhancement in each region was measured in linear arbitrary enhancement units (AEU). A time–intensity curve was generated for each region of interest and for each examination the below parameters were calculated [46].

- Peak enhancement (expressed in AEU): enhancement curves were produced after measurement of intensity by means of Vivid-I software (General Electric) and dividing by the maximum value of intensity.
- Time to peak (s): calculated from injection of contrast agent to peak intensity.
- Time to wash-out (s): calculated from injection of contrast agent to return to baseline.
- Total enhancement time (s): calculated from beginning of enhancement to return to baseline.
- Wash-in time (s): calculated from beginning of enhancement to peak intensity.
- Wash-out time (s): calculated from peak intensity to return to baseline.

#### 4.5.5. Modelling for Analysis of Infection Results

In the study, there was a difficulty with attempts to estimating incidence rate (new ‘infection’ per animal at risk for each time point at risk), because, in many cases, the site under study might change from being ‘infected’ to being ‘uninfected’ and vice-versa; therefore, when there was a long time-interval between sampling points, it was not possible to know what happened between the two sampling points (i.e., how many infections and ‘cures’ there might have occurred). Therefore, the following definitions were initially made [47].

- ‘Isolation of bacteria’ was equivalent to ‘infection with’; ‘isolation of bacteria from the swab’ was equivalent to ‘infection of the anterior part of the vagina’ and ‘isolation of bacteria from uterine tissue sample’ was equivalent to ‘infection of uterus’.
- On a particular sampling point, a sampling site (anterior part of the vagina, uterus) was defined as being ‘at risk of becoming infected’ (i.e., becoming bacteriologically positive) if it had been uninfected (i.e., bacteriologically negative) on the previous sampling point. On the subsequent sampling point, this sampling site (anterior part of the vagina, uterus) could be either ‘infected’ (in which case it was not at risk) or ‘uninfected’ (in which case it was still at risk). On subsequent sampling points, if this site was ‘uninfected’, then it was again ‘at risk’.
- If a sampling site was infected on one sampling point but not on the next one, then the infection was deemed to have been eliminated half-way between the two sampling points; conversely, if a sampling site was uninfected on one sampling point and infected on the next one, then the infection was deemed to have taken place half way between the two dates.
- If a sampling site was infected with the same organism on two consecutive samplings, then it was considered to have been infected throughout the period between those two sampling points; conversely, if a sampling site was uninfected on two consecutive samplings, then it was uninfected throughout the time between those two sampling points.
- Recurrence of infection was defined as re-isolation of an organism from a previously infected sampling site, after a sampling, in which no isolation of any organism took place in-between two samplings.

Based on the above, it was possible to calculate an estimate of the length of time a sampling site was at risk before it became infected, as well as the length of time of each infection. Sampling sites contributed more than one value, if recurrence of infection had occurred.

#### 4.5.6. Evaluation of Cellular Infiltration

Smears from vaginal swab samples were evaluated to assess leucocyte subpopulations, by means of semi-quantitative observational method using the 40× objective lens of a Zeiss-Axiostar Microscope (Carl Zeiss, Göttingen, Germany) with a 10× eyepiece lens. In each slide, at least 50 fields were observed and at least 100 leucocytes were counted.

Uterine tissue samples were evaluated as above and 50 fields were observed on each slide. A score was assigned for average number ($\bar{x}$) of leucocytes per field therein as follows; 0: $\bar{x}$ < 1 leucocyte, 1: 1 ≤ $\bar{x}$ < 5 leucocytes, 2: 5 ≤ $\bar{x}$ < 10 leucocytes, 3: 10 ≤ $\bar{x}$ < 15 leucocytes, 4: $\bar{x}$ ≥ 15 leucocytes per field.

#### 4.5.7. Measures for Reproductive Performance

The following measures of reproductive performance were calculated [31].

- Mating rate: number of ewes mated by rams during the whole reproductive period/number of ewes exposed to the ram × 100.
- Pregnancy rate: number of ewes that were found pregnant at ultrasonographic examination 50 and 100 days after ram introduction/number of ewes exposed to the ram × 100.
- Abortion rate: number of ewes that aborted before the 140th day of gestation/number of ewes exposed to the ram × 100.
- Lambing rate: number of ewes that lambed/number of ewes exposed to rams × 100.
- Total lambs per ewe: number of liveborn and stillborn lambs/number of ewes that lambed.
- Stillbirth rate: number of stillborn lambs/number of liveborn and stillborn lambs × 100.

#### 4.5.8. Statistical Analysis

Data were entered into Microsoft Excel for analysis. Basic descriptive analysis was performed. The outcomes of interest were considered.

Comparisons of frequencies of clinical signs and bacterial isolations for I versus C group were performed in a table of cross-categorised frequency data by use of Pearson chi-square test or Fisher-exact test, as appropriate. Time of first appearance of an outcome and duration of outcome under evaluation were compared between groups as above by means of Mann-Whitney test. The Kruskal-Wallis test and the Mann-Whitney tests were used to evaluate differences in bacterial isolations between post-partum stages.

For ultrasonographic measurements (B-mode, Doppler mode), repeated measures mixed effect linear regression models were used to determine whether outcomes changed over the course of the study period. Fixed effect was the time-point of the study (i.e., L0, L1, etc.). Effect of experimental subjects (animals) was included as random effect in the model. Models were adjusted for repeated measures within animals. Initially, separate analyses were performed for each stage post-lambing (S_1_–S_4_), which were followed by an analysis that took into account all measurements (18 time-points) carried out. The same method was also used for analysing results of haematological examinations.

For ultrasonographic (B-mode, Doppler mode) and haematological parameters, as well for proportions of types of leucocytes in vaginal samples, analysis of covariance was performed between ewes in the two groups (I and C). In this analysis, measurements obtained after challenge were compared between groups after taking into account and eliminating a possible effect of measurements made before challenge.

For CEUS results, repeated measures mixed effect linear regression models were used to study outcomes over the measurement period; the effect of animals was included as random effect in the model, which was adjusted for repeated measures within animals and comparisons were made between inoculated and control ewes.

The Mann-Whitney test was used to evaluate differences between groups in scores for average leucocyte numbers in uterine tissue samples. Results of reproductive performance were evaluated by comparison of proportions or Student’s t-test, as appropriate per type of result.

In all cases, level of significance was set at *p* = 0.05.

## Figures and Tables

**Table 1:** Post-partum echogenicity: ratio GS_C_/GS_E_^1^ (mean ± standard error of the mean) in the uterus of ewes with experimentally induced uterine infection (group I) or of uninoculated controls (group C).

		Stage Post-Partum ^2^			
		S_1_	S_2_	S_3_	S_4_
**Group**	I	0.94 ± 0.06 ^a,m^	1.00 ± 0.04 ^n^	1.22 ± 0.05 ^b,m,n^	0.88 ± 0.04
	C	0.84 ± 0.04 ^a,m,n^	0.97 ± 0.04 ^m^	0.97 ± 0.04 ^b,n^	0.93 ± 0.02

^a,b^: same superscript within a column indicates significant difference between I and C groups (^a^: 0.05 < *p* ≤ 0.01, ^b^: *p* < 0.01)—^m,n^: same superscripts within a row indicate significant difference between stages (*p* < 0.01). ^1^ GS_C_: Grey-scale of caruncular tissue, GS_E_: Grey-scale of endometrium. ^2^ S_1_: included samples collected before inoculation, on L0 and L1, S_2_: included samples collected after challenge up to and including D4, S_3_: included samples collected from D6 to D12 and S_4_: included samples collected from D17 to D62 (D0: day of inoculation, which took place on the 1st day post-partum).

**Table 2:** Post-partum ultrasonographically measured dimensions (mean ± standard error of the mean) of uterine structures of ewes with experimentally induced uterine infection (group I) or of uninoculated controls (group C).

				Stage Post-Partum ^1^			
				S_1_	S_2_	S_3_	S_4_
**Uterine dimensions**	diameter of caruncles (cm)	**Group**	I	1.44 ± 0.03 ^m,n,o^	1.32 ± 0.02 ^b,m,p,q^	1.15 ± 0.05 ^b,n,p^	0.94 ± 0.14 ^o,q^
			C	1.37 ± 0.03 ^m,n,o^	1.23 ± 0.02 ^b,m,p,q^	0.99 ± 0.04 ^b,n,p,r^	0.77 ± 0.02 ^o,q,r^
	myometrial thickness (cm)		I	0.48 ± 0.01 ^m,n,o,n^	0.40 ± 0.01 ^m,p,q^	0.31 ± 0.01 ^a,n,p,r^	0.23 ± 0.01 ^a,o,q,r^
			C	0.47 ± 0.01 ^m,n,o^	0.39 ± 0.01 ^m,p,q^	0.29 ± 0.01 ^a,n,p,r^	0.22 ± 0.00 ^a,o,q,r^
	endometrium thickness (cm)		I	0.72 ± 0.06 ^m,n,o^	0.58 ± 0.03 ^m,p,q^	0.44 ± 0.05 ^n,p,r^	0.25 ± 0.02 ^o,q,r^
			C	0.64 ± 0.03 ^m,n,o^	0.54 ± 0.02 ^m,p,q^	0.44 ± 0.03 ^n,p,r^	0.28 ± 0.01 ^o,q,r^
	diameter of uterine lumen (cm)		I	0.62 ±0.06 ^m,n,o^	0.50 ± 0.02 ^m,p,q^	0.36 ± 0.01 ^n,p,r^	0.24 ± 0.01 ^o,q,r^
			C	0.63 ± 0.05 ^m,n,o^	0.46 ± 0.02 ^m,p,q^	0.35 ± 0.01 ^n,p,r^	0.23 ± 0.01 ^o,q,r^

^a,b^: with reference to the same parameter, same superscript within a column indicates significant difference between I and C groups (^a^: 0.01 ≤ *p* < 0.05, ^b^: *p* < 0.01)—^m–r^: same superscripts within a row indicate significant difference between stages (*p* < 0.035). ^1^ S_1_: included samples collected before inoculation, on L0 and L1, S_2_: included samples collected after challenge up to and including D4, S_3_: included samples collected from D6 to D12 and S_4_: included samples collected from D17 to D62 (D0: day of inoculation, which took place on the 1st day post-partum).

**Table 3:** Mean daily reduction of dimensions of uterine structures, as calculated based on ultrasonographic measurements, of ewes with experimentally induced uterine infection (group I) or of uninoculated controls (group C).

				Stage Post-Partum ^1^		
				S_2_	S_3_	S_4_
**Uterine dimensions**	diameter of caruncles (cm)	**Group**	I	0.034	0.017 ^a^	0.009
			C	0.042	0.027 ^a^	0.008
	myometrial thickness (cm)		I	0.068	0.030	0.007
			C	0.067	0.033	0.007
	endometrium thickness (cm)		I	0.101	0.155 ^b^	0.138 ^b^
			C	0.092	0.018 ^b^	0.007 ^b^
	diameter of uterine lumen (cm)		I	0.127 ^a^	0.142 ^b^	0.130 ^b^
			C	0.076 ^a^	0.044 ^b^	0.012 ^b^

^a,b^: with reference to the same parameter, same superscript within a column indicates significant difference between I and C groups (^a^: 0.01 ≤ *p* < 0.05, ^b^: *p* < 0.01). ^1^ S_1_: included samples collected before inoculation, on L0 and L1, S_2_: included samples collected after challenge up to and including D4, S_3_: included samples collected from D6 to D12 and S_4_: included samples collected from D17 to D62 (D0: day of inoculation, which took place on the 1st day post-partum).

**Table 4:** Post-partum Doppler ultrasonographic measurements (mean ± standard error of the mean) in the uterine artery of ewes with experimentally induced uterine infection (group I) or of uninoculated controls (group C).

				Stage Post-Partum ^1^			
				S_1_	S_2_	S_3_	S_4_
**Doppler ultrasonographic parameter**	uterine artery diameter (cm)	**Group**	I	0.44 ± 0.02 ^m,n,o^	0.38 ± 0.01 ^b,m,p,q^	0.28 ± 0.01 ^b,n,p,r^	0.22 ± 0.01 ^c,o,q,r^
			C	0.44 ± 0.02 ^m,n,o^	0.35 ± 0.01 ^b,m,p,q^	0.25 ± 0.01 ^b,n,p,r^	0.18 ± 0.01 ^c,o,q,r^
	resistance index		I	0.70 ± 0.02 ^m,n,o^	0.77 ± 0.01 ^c,m,p^	0.81 ± 0.02 ^n,q^	0.86 ± 0.02 ^o,p,q^
			C	0.74 ± 0.02 ^m,n,o^	0.81 ± 0.01 ^c,m,p^	0.84 ± 0.02 ^n^	0.85 ± 0.02 ^o,p^
	pulsatility index		I	1.50 ± 0.12 ^a,m,n,o^	1.98 ± 0.08 ^c,m,p^	2.18 ± 0.11 ^b,n,q^	2.81 ± 0.17 ^o,p,q^
			C	1.79 ± 0.10 ^a,m,n,o^	2.45 ± 0.09 ^c,m,p^	2.58 ± 0.15 ^b,n,q^	2.99 ± 0.13 ^o,p,q^
	systolic:diastolic velocity ratio		I	4.27 ± 0.74 ^m,n^	5.88 ± 0.86 ^o^	6.93 ± 0.73 ^m,p^	10.70 ± 1.96 ^n,o,p^
			C	4.27 ± 0.30 ^m,n,o^	6.84 ± 0.45 ^m,p^	7.44 ± 0.69 ^n,q^	12.91 ± 1.57 ^o,p,q^
	general pressure (mm Hg)		I	1.22 ± 0.24 ^m,n^	0.87 ± 0.06 ^o^	0.72 ± 0.07 ^m,p^	0.53 ± 0.07 ^n,o,p^
			C	1.09 ± 0.19 ^m,n^	0.82 ± 0.07 ^o^	0.71 ± 0.08 ^p^	0.43 ± 0.02 ^n,o,p^
	mean pressure (mm Hg)		I	0.40 ± 0.08 ^m,n,o^	0.22 ± 0.02 ^b,m,p^	0.20 ± 0.03 ^n,q^	0.10 ± 0.02 ^o,p,q^
			C	0.26 ± 0.03 ^m,n,o^	0.16 ± 0.02 ^b,m,p^	0.14 ± 0.02 ^n,q^	0.07 ± 0.01 ^o,p,q^
	mean velocity (m s^−1^)		I	0.73 ± 0.05	0.69 ± 0.02	0.71 ± 0.03	0.73 ± 0.03
			C	0.67 ± 0.03	0.69 ± 0.02	0.74 ± 0.04	0.69 ± 0.02
	systolic acceleration (m s^−1^)		I	6.60 ± 1.26	6.02 ± 0.38 ^m^	8.95 ± 1.15 ^m^	6.68 ± 0.72
			C	6.89 ± 0.75 ^m^	6.67 ± 0.30 ^n,o,p^	8.89 ± 0.69 ^m,o^	7.78 ± 0.45 ^n,p^
	blood flow volume (mL min^−1^)		I	242.1 ± 31.4 ^m,n,o^	144.7 ± 10.6 ^c,m,p,q^	68.9 ± 8.3 ^c,n,p,r^	28.2 ± 3.3 ^b,o,q,r^
			C	209.1 ± 25.2 ^m,n,o^	105.5 ± 9.6 ^c,m,p,q^	43.9 ± 3.8 ^c,n,p,r^	17.0 ± 1.3 ^b,o,q,r^

^a–c^: with reference to the same parameter, same superscripts within a column indicate significant difference between A and C groups (^a^: 0.025 < *p* ≤ 0.050, ^b^: 0.01 < *p* ≤ 0.025, ^c^: *p* ≤ 0.01); ^m–r^: same superscripts within a row indicate significant difference between stages (*p* < 0.045). ^1^ S_1_: included samples collected before inoculation, on L0 and L1, S_2_: included samples collected after challenge up to and including D4, S_3_: included samples collected from D6 to D12 and S_4_: included samples collected from D17 to D62 (D0: day of inoculation, which took place on the 1st day post-partum).

**Table 5:** Quantitative results (median) of contrast-enhanced ultrasonographic examination of the uterus of ewes with experimentally induced uterine infection (group I) or of uninoculated controls (group C).

	Region of Interest	
Ultrasonographic Parameter	Caruncular Tissue	Intercaruncular Area
Group I		
Peak enhancement (AEU)	42.934 ^a,b^	118.359 ^b^
Time to peak (s)	48	52
Time to wash-out (s)	78	128
Total enhancement time (s)	64	122
Wash-in time (s)	34	46
Wash-out time (s)	30	76
Group C		
Peak enhancement (AEU)	99.947 ^a,b^	118.565 ^b^
Time to peak (s)	58	70
Time to wash-out (s)	138	164
Total enhancement time (s)	130	150
Wash-in time (s)	50	56
Wash-out time (s)	80	94

^a^: same superscript within a column indicates significant difference between I and C groups (*p* < 0.001); ^b^: same superscript within a row indicates significant difference the two tissues imaged (*p* < 0.01).

**Table 6:** Frequency of isolation of bacteria from anterior vagina samples of ewes with experimentally induced uterine infection (group I) or of uninoculated controls (group C).

	Stage Post-Partum ^1^			
Group	S_1_	S_2_	S_3_	S_4_
Isolation of any bacterial species				
I	3/24 ^2^	50/60	28/30	48/70
C	3/24	56/72	28/36	56/84
Isolation of *E. coli*				
I	2/24	48/60	27/30	34/70
C	2/24	39/72	20/36	37/84

^1^ S_1_: included samples collected before inoculation, on L0 and L1, S_2_: included samples collected after challenge up to and including D4, S_3_: included samples collected from D6 to D12 and S_4_: included samples collected from D17 to D62 (D0: day of inoculation, which took place on the 1st day post-partum). ^2^ m/n = samples that yielded bacteria/total number of samples collected.

**Table 7:** Proportions (%) of leucocyte types observed in vaginal swab samples of ewes with experimentally induced uterine infection (group I) or of uninoculated controls (group C).

				Stage Post-Partum ^1^			
				S_1_	S_2_	S_3_	S_4_
**Leucocyte types**	Neutrophils	**Group**	I	98.7 ± 0.9 ^m,n,o^	94.0 ± 1.5 ^c,m,p^	87.0 ± 4.6 ^a,n,p,q^	66.7 ± 5.0 ^a,o,p,q^
			C	98.6 ± 0.7 ^m,n^	98.6 ± 0.3 ^c,o,p^	95.9 ± 1.0 ^a,m,o,q^	79.6 ± 4.6 ^a,n,p,q^
	Lymphocytes		I	1.3 ± 1.0 ^a,m,n,o^	5.3 ± 1.5 ^c,m^	9.1 ± 3.6 ^n^	8.2 ± 2.1 ^o^
			C	0.7 ± 0.4 ^m,n^	1.0 ± 0.2 ^c,o,p^	3.5 ± 1.0 ^m,o^	7.2 ±2.6 ^n,p^
	Macrophages		I	0.0 ± 0.0 ^a,m^	0.8 ± 0.4 ^m^	0.5 ± 0.3	0.8 ± 0.7
			C	0.7 ±0.4 ^a^	0.4 ± 0.1	0.6 ± 0.3	0.8 ± 0.4

^a,b^: with reference to the same parameter, same superscripts within a column indicate significant difference between A and C groups (^a^: 0.025 < *p* < 0.05, ^b^: 0.01 < *p* < 0.025, ^c^: *p* ≤ 0.01); ^m–r^: same superscripts within a row indicate significant difference between stages (*p* < 0.03). ^1^ S_1_: included samples collected before inoculation, on L0 and L1, S_2_: included samples collected after challenge up to and including D4, S_3_: included samples collected from D6 to D12 and S_4_: included samples collected from D17 to D62 (D0: day of inoculation, which took place on the 1st day post-partum).

**Table 8:** Haematological results (mean ± standard error of the mean) in samples of ewes with experimentally induced uterine infection (group I) or of uninoculated controls (group C).

	Stage Post-Partum ^1^			
Group	S_1_	S_2_	S_3_	S_4_
Erythrocyte counts (×10^6^ cells μL^−1^)				
I	9.02 ± 0.29	8.98 ± 0.18 ^c^	9.00 ± 0.26	8.91 ± 0.16 ^c^
C	8.58 ± 0.35	8.38 ± 0.18 ^c^	8.62 ± 0.26	8.16 ± 0.14 ^c^
Haematocrit (%)				
I	30.5 ± 1.0	30.2 ± 0.6	30.0 ± 0.9	29.1 ± 0.5 ^c^
C	30.1 ± 1.2 ^m^	29.5 ± 0.5 ^n^	29.7 ± 0.7 ^o^	27.5 ± 0.4 ^c,m,n,o^
Haemoglobin concentration (g dL^−1^)				
I	9.94 ± 0.29	9.97 ± 0.16 ^m^	9.85 ± 0.22	9.61 ± 0.14 ^c,m^
C	9.73 ± 0.38 ^m^	9.63 ± 0.18 ^n^	9.61 ± 0.26 ^o^	8.92 ± 0.13 ^c,m,n,o^
Mean corpuscular volume (fL)				
I	34.0 ± 0.8	33.8 ± 0.4 ^c,m^	33.4 ± 0.5	32.7 ± 0.3 ^c,m^
C	35.2 ± 0.6 ^m^	35.4 ± 0.3 ^c,n^	34.6 ± 0.5	34.0 ± 0.3 ^c,m,n^
Mean corpuscular haemoglobin concentration (g dL^−1^)				
I	32.9 ± 0.8	33.3 ± 0.4	33.2 ± 0.5	33.3 ± 0.3 ^c^
C	32.4 ± 0.2	32.7 ± 0.1	32.4 ± 0.2	32.4 ± 0.2 ^c^
Total leucocyte counts (cells μL^−1^)				
I	12696 ± 1126 ^m,n,o^	10200 ± 421 ^m^	10512 ± 445 ^n^	10247 ± 279 ^o^
C	12901 ± 856 ^m,n,o^	10938 ± 411 ^m,p^	10226 ± 404 ^n^	9886 ± 288 ^o,p^
Neutrophil counts (cells μL^−1^)				
I	7412 ± 1039 ^m,n,o^	4648 ± 410 ^c,m,p^	4596 ± 278 ^n,q^	3760 ± 175 ^o,p,q^
C	8275 ± 692 ^m,n,o^	5867 ± 303 ^c,m,p,q^	4649 ± 270 ^n,p,r^	4031 ± 153 ^o,q,r^
Lymphocyte counts (cells μL^−1^)				
I	4382 ± 361 ^m,n^	4773 ± 194 ^b,o,p^	5376 ± 269 ^b,m,o,p^	5820 ± 169 ^c,n,p,q^
C	3918 ± 224 ^m,n^	4227 ± 136 ^b,o,p^	4746 ± 244 ^b,m,o^	4860 ± 146 ^c,n,p^
Monocyte counts (cells μL^−1^)				
I	170 ± 31 ^c,m,n^	237 ± 23 ^c,m^	191 ± 26 ^c^	240 ± 16 ^a,n^
C	291 ± 38 ^c^	354 ±21 ^c,m^	374 ± 34 ^c,n^	293 ± 22 ^a,m,n^
Eosinophil counts (cells μL^−1^)				
I	271 ± 42 ^m^	200 ± 13 ^c^	188 ± 27 ^c,m^	213 ± 17 ^c^
C	270 ± 42 ^m^	338 ± 22 ^c,n^	301 ± 34 ^c,o^	547 ± 68 ^c,m,n,o^
Basophil counts (cells μL^−1^)				
I	77 ± 8 ^m^	82 ± 4 ^n^	90 ± 9 ^o^	111 ± 6 ^m,n,o^
C	76 ± 7 ^m^	86 ± 3 ^n^	83 ± 5 ^o^	103 ± 7 ^m,n,o^
Thrombocyte counts (×10^3^ cells μL^−1^)				
I	509 ± 19 ^c,m,n,o^	552 ± 14 ^c,m,p^	668 ± 25 ^c,n,p,q^	574 ± 16 ^c,o,q^
C	669 ± 49 ^c,m^	747 ± 28 ^c^	826 ± 47 ^c,m,n^	727 ± 31 ^c,n^

^a–c^: with reference to the same parameter, same superscripts within a column indicate significant difference between A and C groups (^a^: 0.025 < *p* ≤ 0.050, ^b^: 0.01 < *p* ≤ 0.025, ^c^: *p* ≤ 0.01); ^m–r^: same superscripts within a row indicate significant difference between stages (*p* < 0.045). ^1^ S_1_: included samples collected before inoculation, on L0 and L1, S_2_: included samples collected after challenge up to and including D4, S_3_: included samples collected from D6 to D12 and S_4_: included samples collected from D17 to D62 (D0: day of inoculation, which took place on the 1st day post-partum).

**Table 9:** Reproductive performance, during the subsequent breeding season, of ewes with experimentally induced uterine infection (group I) or of uninoculated controls (group C).

	Group	
Measure of Reproductive Performance	I	C
Mating rate	100%	100%
Pregnancy rate	100%	100%
Abortion rate	0%	0%
Lambing rate	100%	100%
Total lambs per ewe	1.90	2.00
Stillbirth rate	0%	0%

## References

[1] Noakes D.E., Noakes D.E., Parkinson T.J., England G.C.W. (2009). The puerperium and the care of the newborn. Arthur’s Veterinary Reproduction and Obstetrics.

[2] Badawi M.E., Makawi S.E.A., Abdelghafar R.M., Ibrahim M.T. (2014). Assessment of postpartum uterine involution and progesterone profile in Nubian goats (*Capra hircus*). J. Adv. Vet. Anim. Res..

[3] Hayder M., Ali A. (2008). Factors affecting the postpartum uterine involution and luteal function of sheep in the subtropics. Small Rumin. Res..

[4] Fernandes C.E., Cigerza C.F., Pinto G.D.S., Miazi C., Barbosa-Fereira M., Martins C.F. (2013). Parturition characteristics and uterine involution in native sheep from Brazilian Pantanal. Cienc. Anim. Bras..

[5] Ioannidi K.S., Mavrogianni V.S., Valasi I., Barbagianni M.S., Vasileiou N.G.C., Amiridis G.S., Fthenakis G.C., Orfanou D.C. (2017). Ultrasonographic examination of the uterus of ewes during the post-partum period. Small Rumin. Res..

[6] Palmieri C., Schiavi E., Salda L.D. (2011). Congenital and acquired pathology of ovary and tubular genital organs in ewes: A review. Theriogenology.

[7] Mavrogianni V.S., Brozos C. (2008). Reflections on the causes and the diagnosis of peri-parturient losses of ewes. Small Rumin. Res..

[8] Sargison N. (2008). Sheep Flock Health A Planned Approach.

[9] Tzora A., Leontides L.S., Amiridis G.S., Manos G., Fthenakis G.C. (2002). Bacteriological and epidemiological findings during examination of the uterine content of ewes with retention of fetal membranes. Theriogenology.

[10] Hussain S.O., Al-Zubaili S.F., Asofi M. (2013). Different endometritis treatments in ewe: Comparative study. J. Agric. Vet. Sci..

[11] Zhao W., Caro F., Robins W., Mekalanos J.J. (2018). Antagonism toward the intestinal microbiota and its effect on *Vibrio cholera* virulence. Science.

[12] Sana T.G., Flaugnatti N., Lugo K.A., Lam L.H., Jacobson A., Baylot V., Durand E., Journet L., Cascales E., Monck D.M. (2016). *Salmonella Typhimurium* utilizes a T6SS-mediated antibacterial weapon to establish in the host gut. Proc. Nat. Acad. Sci. USA.

[13] Navarro-Garcia F., Ruiz-Perez F., Cataldi Á., Larzábal M. (2019). Type VI secretion system in pathogenic *Escherichia coli*: Structure, role in virulence, and acquisition. Front. Microbiol..

[14] Faigl V., Keresztes M., Márton A., Fébel H., Kulcsár M., Nagy S., Cseh S., Solti L., Hyszenicza G. (2011). Effect season and photoperiod on the time of first postpartum ovulation in Awassi ewes. Acta Vet. Hung..

[15] Cai T.Q., Weston P.G., Lund L.A., Brodie B., McKenna D.J., Wagner W.C. (1994). Association between neutrophil functions and periparturient disorders in cows. Am. J. Vet. Res..

[16] Leung S.T., Derecka K., Mann G.E., Flint A.P.F., Wathes D.C. (2000). Uterine lymphocyte distribution and interleukin expression during early pregnancy in cows. J. Reprod. Fertil..

[17] Brodzki P., Kostro K., Brodzki A., Lisiecka U., Kurek Ł., Marczuk J. (2014). Phenotyping of leukocytes and granulocyte and monocyte phagocytic activity in the peripheral blood and uterus of cows with endometris. Theriogenology.

[18] Singh J., Murray R.D., Mshelia G., Woldehiwet Z. (2008). The immune status of the bovine uterus during the peripartum period. Vet. J..

[19] Ramadan A.A., Johnson G.L., Lewis G.S. (1997). Regulation of uterine immune function during the estrous cycle and in response to infectious bacteria in sheep. J. Anim. Sci..

[20] Adegboyega P.A., Pei Y., McLarty J. (2010). Relationship between eosinophils and chronic endometritis. Hum. Pathol..

[21] Bondurant R.H. (1999). Inflammation in the bovine female reproductive tract. J. Anim. Sci..

[22] Ababneh M.M., Degefa T. (2005). Ultrasonic assessment of puerperal uterine involution in Balaby goats. J. Vet. Med..

[23] Barlund C.S., Carruthers T.D., Waldner C.L., Palmer C.W. (2008). A comparison of diagnostic techniques for postpartum endometritis in dairy cattle. Theriogenology.

[24] Meira E.B.S., Henriques L.C.S., Sá L.R.M., Gregory L. (2012). Comparison of ultrasonography and histopathology for the diagnosis of endometritis in Holstein-Friesian cows. J. Dairy Sci..

[25] Kasimanickam R., Duffield T.F., Foster R.A., Gartley C.J., Leslie K.E., Walton J.S., Johnson W.H. (2004). Endometrial cytology and ultrasonography for the detection of subclinical endometritis in postpartum dairy cows. Theriogenology.

[26] Derar D.R., Hayder M., Ali A., Hamdoun H. (2012). Postpartum uterine involution and luteal activity in Farafra ewes lambing in autumn. Assiut Vet. Med. J..

[27] Fasulkov I. (2014). Ultrasonography of uterine involution in goats. J. Fac. Vet. Med. Istanb. Univ..

[28] Medan M.S., El-Daek T. (2015). Uterine involution and progesterone level during the postpartum period in Barbady ewes in north Libya. Open Vet. J..

[29] Gray C.A., Stewart M.D., Johnson G.A., Spencer T.E. (2003). Postpartum uterine involution in sheep: Histoarchitecture and changes in endometrial gene expression. Reproduction.

[30] Hurley J.V. (1983). Acute Inflammation.

[31] Fthenakis G.C. (2004). Effects of retention of fetal membranes on subsequent reproductive performance of dairy ewes. Theriogenology.

[32] Valasi I., Barbagianni M.S., Ioannidi K.S., Vasileiou N.G.C., Fthenakis G.C., Pourlis A. (2017). Developmental anatomy of sheep embryos, as assessed by means of ultrasonographic evaluation. Small Rumin. Res..

[33] Fthenakis G.C., Arsenos G., Brozos C., Fragkou I.A., Giadinis N.D., Giannenas I., Mavrogianni V.S., Papadopoulos E., Valasi I. (2012). Health management of ewes during pregnancy. Anim. Reprod. Sci..

[34] Amiridis G.S., Fthenakis G.C., Dafopoulos J., Papanikolaou T., Mavrogianni V.S. (2003). Use of cefquinome for prevention and treatment of bovine endometritis. J. Vet. Pharmacol. Ther..

[35] Mavrogianni V.S., Amiridis G.S., Gougoulis D.A., Fragkou I.A., Fthenakis G.C. (2007). Efficacy of difloxacin for the control of postpartum uterine infections of ewes. J. Vet. Pharmacol. Ther..

[36] Miles A.A., Misra J.S. (1938). The estimation of the bactericidal power of the blood. J. Hyg. Camb..

[37] Petridis I.G., Barbagianni M.S., Ioannidi K.S., Samaras E., Fthenakis G.C., Vloumidi E.I. (2017). Doppler ultrasonographic examination in sheep. Small Rumin. Res..

[38] Galatos A.D. (2011). Anesthesia and analgesia in sheep and goats. Vet. Clin. N. Am. Food Anim. Pract..

[39] Barrow G.I., Feltham R.K.A. (1993). Manual for the Identification of Medical Bacteria.

[40] Euzeby J.P. (1997). List of bacterial names with standing in nomenclature: A folder available on the Internet. Int. J. Syst. Bacteriol..

[41] National Institutes of Health (2013). ImageJ: Image Processing and Analysis in Java. http://rsbweb.nih.gov/ij/.

[42] Ojala T., Pietikaeinen M., Maeenpaea T. (2002). Multiresolution gray-scale and rotation invariant texture classification with local binary patterns. IEEE Trans. Pattern Anal..

[43] Maulik D., Maulik D. (2005). Spectral Doppler sonography: Waveform analysis and hemodynamic information. Doppler Ultrasound in Obstetrics and Gynecology.

[44] Ginther O.J. (2007). Ultrasonic Imaging and Animal Reproduction: Color-Doppler Ultrasonography.

[45] Wood M., Romine L.E., Lee Y.K., Richman K.M., O’Boyle N.M.K., Paz D.A., Chu P.K., Pretorius D.H. (2010). Spectral Doppler signature waveforms in ultrasonography: A review of normal and abnormal waveforms. Ultrasound Q..

[46] Mantziaras G., Vasileiou N.G.C., Ioannidi K.S., Mavrogianni V.S., Gougoulis D.A., Fthenakis G.C., Petridis I.G., Barbagianni M.S. (2018). Use of contrast-enhanced ultrasonographic examination to evaluate health status of mammary glands of ewes at the end of a lactation period. J. Dairy Res..

[47] Mavrogianni V.S., Cripps P.J., Fthenakis G.C. (2007). Bacterial flora and risk of infection of the ovine teat duct and mammary gland throughout lactation. Prev. Vet. Med..

